# Relationship between Adiponectin and Blood Pressure in Obese Latino Adolescent Boys with a Family History of Type 2 Diabetes

**DOI:** 10.21203/rs.3.rs-3101635/v1

**Published:** 2023-08-03

**Authors:** Kristin Hijazin, Brandon Smith, Coleby Garrett, Allan Knox, Louise A. Kelly

**Affiliations:** California Lutheran University

**Keywords:** Latino youth, Pediatric, Males, Adiponectin, Blood Pressure, Obesity

## Abstract

**INTRODUCTION:**

Adipokines are associated with several pathological states including, metabolic syndrome, obesity, insulin resistance and type 2 diabetes. One of these adipokines, adiponectin is of particular interest as it has been shown to have numerous anti-inflammatory effects, However, the association between adiponectin and blood pressure remains inconclusive especially in the obese Hispanic adolescent.

**PURPOSE:**

to investigate the relationship between plasma adiponectin and blood pressure in obese Latino adolescents’ boys with a family history of Type 2 diabetes.

**METHODS:**

Thirty two obese Latino male adolescents aged 14–17 years with a family history of type 2 diabetes underwent a frequently sampled glucose tolerance test (FSIVGTT) to measure insulin sensitivity. Body composition was assessed using dual energy x-ray absorptiometry. Obesity was defined as having a BMI percentile ^3^95. Blood pressure was assessed using the Dinamap automated blood pressure monitor, and the average of three readings was used in the analysis. Fasting plasma adiponectin was determined using radioimmunoassay.

**RESULTS:**

There was a strong positive significant correlation for adiponectin and Systolic blood pressure(SBP) (p< 0.027) and a moderate, positive significant correction for Diastolic blood pressure(DBP) (p< 0.028). A multivariate liner regression showed that plasma adiponectin could significantly predict 19% of the variance in SBP (p=0.017, and 33% for DBP (p=0.017).

**CONCLUSION:**

In conclusion, adiponectin was positively and significantly correlated to blood pressure in obese Latino adolescent youth. Future studies should investigate this relationship in a large sample of Latino adolescent youth.

## INTRODUCTION

Pediatric obesity is one of the most pertinent health issues of the last century. In 2019, 340 million children aged 5–19 years are considered overweight/obese (1, 2) with a disproportionate impact in racial and ethnic minorities, particularly Latino youth (3)The 2011–2020 National Health and Nutrition Examination Survey (NHANES) shows an increased trend of obesity(95th percentile for age and gender) in Latino youth when compared to other ethnic groups of the same age from 21.8–27.0%; P for trend = 0.006 (4). Studies have also shown that the prevalence of obesity is significantly higher in Latino males. Furthermore, pediatric obesity is commonly associated with several non-communicable diseases such as type 2 diabetes (5), metabolic syndrome (6), fatty liver disease (7), cardiovascular disease (8), several cancers (9) and hypertension (10–12).

Low insulin sensitivity (IS) is also a predictor of high blood pressure (BP), particularly in minority youth (13). In adult and pediatric studies, hypertension has been associated with insulin resistance and hyperinsulinemia (13–15). In a meta-analysis by Wang and colleagues the relationship between insulin resistance and hypertension was investigated in 11 studies, involving 10,230 participants. The results of this analysis suggested that insulin resistance is independently associate with hypertension in the general population (16).

The prevalence of hypertension in children is threefold higher in obese children than in non-obese children (12). A systemic review of 22 articles by Lona et al., concluded children with higher BP’s and BMIs are related to increased cardiovascular biomarker central pulse wave velocity (cPWV) (17). A higher blood pressure (BP) is also associated with a higher body mass index (BMI) (18), both of which are higher in children from minority backgrounds compared with their white counterparts (19). In addition, changes in secretion of cytokines such as adiponectin have been linked to cardiovascular risk factors through the effects on insulin sensitivity.

Furthermore, adiponectin, an adipose tissue-derived protein with insulin-sensitizing and antiatherogenic properties, has been associated with the regulation of BP in adults and adolescents (20, 21). During puberty, adiponectin has been shown to decrease in boys (22) and is therefore associated with Type 2 diabetes (23) and metabolic syndrome (24). Therefore, the aim of this study was to investigate the relationship between plasma adiponectin and blood pressure in obese Latino adolescents’ boys with a family history of Type 2 diabetes.

## METHODS AND PROCEDURES

### Participants

Forty-three (*N* = 43) participants were recruited from the greater Los Angeles County area through medical clinics, advertisements, and local schools to participate in the study (Families United for Education and Research for Strong Adolescent Latinos, **FUERSA**). Participants were recruited to the study if they met the following study inclusion criteria: 1) male; 2) grades 9th through 12th (approximately 14–18 years of age); 3) with a BMI ≥95th percentile for age and sex; 4) of Latino ancestry (parents and grandparents descent as determined by self-report); 5) absence of diabetes using established guidelines; 6) absence of comorbid inflammatory disease, secondary hypertension or any condition that would predispose them to type 2 diabetes;7) have a positive family history of type 2 diabetes (determined by parental self-report). The study was conducted in accordance with the guidelines of the Helsinki Declaration. Written informed consent and assent were obtained from the parents and children prior to testing. The Institutional Review Board of the University of Southern California approved the study.

### Anthropometric Measures and Body Composition

Height was measured with a stadiometer to the nearest 0.1cm. Body mass was measured without shoes and in a hospital gown to the nearest 0.05kg using a beam medical scale. Body mass index (BMI) was calculated; age- and sex-specific BMI percentile were determined using *EpiInfo 2000, Version 1.1* (CDC, Atlanta, GA). Obesity was defined as have a BMI ≥95th percentile for age and sex. A dual-energy X-ray absorptiometry (DEXA) scan (Hologic QDR 4500W; Bedford, MA) was performed to estimate total fat mass (FM) and total lean tissue mass (LTM).

### Blood Pressure Measurement

Resting systolic blood pressure (SBP) and diastolic blood pressure (DBP) were measured in the sitting position using a Dinamap automated blood pressure monitor (Critikon Inc., Tampa, FL) with the arm supported at heart level. After sitting quietly for 5 minutes, measurements were obtained on each child using an appropriately sized cuff placed on the right arm. Three readings of blood pressure were obtained, and the average was recorded, according to the recommendations of the American Heart Association (25). Systolic minus diastolic was used to calculate pulse pressure.

### Blood Sampling and Analysis

A venous blood sample was taken after 12 hours overnight of fasting for the following measurements, plasma glucose, and insulin as previously described (26, 27). The insulin resistance index derived by frequently sampled intra venous glucose tolerance test (FSIVGT) was previously described (26, 27). Fasting plasma adiponectin was measured in duplicate using radioimmunoassay (RIA) kits obtained from Linco Research (St. Charles, MO) following the manufacturer’s protocol. The intra- and interassay coefficients of variation were less than 10%.

### Statistical Analyses

All data were checked for normality prior to statistical analysis using descriptive statistics, histograms with normal distribution curves, and the Anderson-Darling (AD) normality tests. Data are presented as means and standard deviation unless indicated otherwise. The correlation analyses in Table 2 and multivariate linear regression analyses were carried out to investigate whether plasma adiponectin could significantly predict SBP and DPB (see Tables 3 and 4). All analysis was conducted using SPSS (version 24 for Mac) with significance or with an alpha > set at 0.05.

## RESULTS

Thirty-two (*N* = 32) obese Latino adolescent males were consented into the study. Characteristics of participants are shown in Table 1.

There were no correlations between SBP and age, height, BMI percentile, waist circumference, hip circumference, waist/hip ratio, fat mass, lean tissue and bone mineral content, disposition index, acute insulin response, and CRP (p > 0.05; see Table 2). Whereas positive nonsignificant relationships were observed between BMI and Weight, pulse pressure, lean tissue, total mass, % fat, % lean, insulin sensitivity, and glucose (p > 0.05; see Table 2). However, adiponectin, showed a strong positively significant correlation with SBP (rho = 0.436, p = 0.018). For DPB, there were weak positive non-significant relationships with age, weight, BMI, pulse pressure, lean tissue, % lean tissue, SI, AIR, and glucose (p > 0.05; see Table 2). Weak negative non-significant relationships were observed between DPB and height, BMI %tile, waist and hip circumference, waist/hip ratio, fat mass, %fat, DI, and CRP (p > 0.05; see Table 2). There was a positive moderate significant correlation between DPB and adiponectin (rho = 0.41, p = 0.02;).

A multivariate linear regression analysis was conducted to investigate whether plasma adiponectin levels could significantly predict SBP and DBP. The results of the regression analysis showed that the model with Adiponectin only could significantly predict 19% of the variance in SBP (R^2^ = (0.192) F (1, 27) = 6.437, p = 0. 017; see Table 3). It was also found that plasma adiponectin predicts SBP (β = 0.439 ± 0.61, p = 0.022). For DPB, the results of the regression analysis showed that the model with Adiponectin only significantly predicted 18% of the variance in DPB (R^2^ = (0.183) F (2, 26) = 5.598, p = 0.026). Plasma adiponectin also predicts DPB (β = 0.428 ± 0.047, p = 0.026). Plasma adiponectin predicted 8% of the variance of pulse pressure (R^2^ = (0.113) F (1, 27) = 3.441, p = 0. 075). However, plasma adiponectin did not significantly predict pulse pressure (β = 0.199 ± 0.107, p = 0.75).

## DISCUSSION

Many adult and pediatric studies have demonstrated a clear relationship between plasma adiponectin and most variables of the metabolic syndrome (28, 29). With, several physiological processes have been proposed to explain the relationship this relationship. These processes include enhanced endothelial function and anti-inflammatory macrophage phenotypes, increased nitric oxide production, in addition to suppressing sympathetic nervous system activity and reduce blood pressure by induce adiponectin secretion (21). However, the relationship between plasma adiponectin and blood pressure is less definitive and can be quite contradictory. To the best of our knowledge this is the first study to investigate the relationship between plasma adiponectin and blood pressure in obese Latino adolescent males with a family history of type 2 diabetes. Our results show a significant positive relationship between plasma adiponectin and blood pressure, with adiponectin significantly predicting blood pressure in obese Latino adolescent males.

A few studies in adults have also shown a significant relationship between adiponectin and blood pressure (30–33). In Pediatrics, data has shown an inverse relationship between adiponectin and blood pressure (34–36), these results are conflicting to those presented in this study, which found a positive relationship between these variables. Most of these pediatric studies investigated this relationship in non-obese, non-Latino (37, 38), a small number were also in obese children (39–41), but none investigated this relationship in pediatric obese Latino males with a family history of type 2 diabetes. A report by Huang et al. showed an inverse relationship between SBP blood pressure but not DBP in a 68 non obese, nondiabetic females. The authors concluded that was independent of any other anthropometric and metabolic variable (38).

Shatat *et al*. also demonstrated in 41 obese and non-obese adolescences with and without the presence of Type 2 Diabetes that adiponectin levels were independently and inversely associated with 24-hr SBP and DBP (41). There results also showed no significant differences in adiponectin by gender, with the authors speculating the results could be attributed to the morbid obesity and its independent effect on reduced adiponectin levels. Because our participants were obese, it seems almost likely that fat distribution may influence adiponectin secretion. Shatat *et al*. study did have a similar sample size of 26 participants to the present study, their participants were male and females, some of their obese participants also were prehypertensive and hypertensive and included 10 black adolescent participants. Furthermore, pathological states such as metabolic syndrome and obesity have been shown to have an association with higher sympathetic nervous system activity. It may be possible that their results differed from ours for these reasons. Preliminary data of more than 100 male adolescents reported by Hunang et al., showed no relationship between adiponectin and BP (38). It is also possible that the relationship between adiponectin and BP may vary by race (42), thus explaining the differences in results from our study with that of Shat et al. African American (42, 43) and Asian Indians have been shown to have lower adiponectin levels when compared to white (44). Zhou et al. investigated the relationship between plasma adiponectin and blood pressure in a very large sample 1300 of children aged 9 to 16 years also found no significant associations (45). In contrast, Mallamaci et al found in a sample of 36 hypertensive and 31 normotensive adults found similar results to our study in that they too found a positive association between plasma adiponectin and blood pressure (46). These and our results suggest that the association between adiponectin and blood pressure may be placing these obese boys with a family history of type 2 diabetes at risk for said disease along with other metabolic and cardiovascular events (47, 48) and an increased risk of future heart failure (49).

There are sever limitations to our study worth noting, firstly, the small sample size; 2) this is a cross-sectional study design, which is not an appropriate design to assess cause and effect between adiponectin and blood pressure. However, the strength of our study is 1) the homogeneous sample, 2) the precise techniques; 3) representative sample of the obese Latino adolescent community and 4) as gender differences were not observed in several studies, we chose to focus on males so as not to confound the analysis.

In conclusion, adiponectin and blood pressure are closely related in these obese adolescent males with a family history of type 2 diabetes. Future larger studies in the Latino obese adolescent population need to be conducted due to the biological importance of adiponectin.

## Figures and Tables

**Table 1:** Characteristics of Participants (n = 32)

Variables	Mean ± SD N = 32
Age (yr)	15.28 ± 1.07
Height (m)	169.47 ± 13.00
Weight (kg)	96.10 ± 15.32
BMI (kg/m^2^)	33.14 ± 4.42
BMI %tile (%)	97.61 ± 2.02
Systolic Blood Pressure (mmHg)	124.61 ± 8.34
Diastolic Blood Pressure (mmHg)	67.12 ± 6.37
Pulse rate (BPM)	70.39 ± 10.77
Waist circumference (m)	0.962 ± 8.46
Hip Circumference (m)	1.083 ± 7.17
Waist/hip ratio	0.89 ± 0.05
Fat (kg)	29.49 ± 8.51
Lean tissue (kg)	63.58 ± 8.18
Lean Tissue and Bone Mineral Content (kg)	66.12 ± 8.39
Total Mass (kg)	95.61 ± 14.05
Fat (%)	30.44 ± 5.93
Lean (%)	66.88 ± 5.69
Insulin Sensitivity (X10^−4^ min^−1^/μU/ml)	2.03 ± 1.47
Acute Insulin Response (μU/ml X 10 min)	1377.75 ± 849.43
Disposition Index (X10^−4^ min^−1^)	2158.26 ± 1193.65
Fasting Glucose (mg/dl)	82.38 ± 9.55
CRP (ng/ml)	1958.11 ± 1982.61
Adiponectin (μg/ml)	7.53 ± 2.54

**Table 2:** Correlations between Adiponectin, anthropometric, body composition, insulin resistance, fasting glucose, CRP and blood pressure.

Variables	Systolic BP		Diastolic BP	
	rho	p	rho	p
Age (yr.)	−0.61	0.76	0.17	0.37
Height (m)	−0.20	0.29	−0.21	0.27
Weight (kg)	0.27	0.16	0.14	0.46
BMI (kg/m^2^)	0.26	0.18	0.18	0.35
BMI %tile (%)	−0.14	0.48	−0.19	0.31
Systolic Blood Pressure (mmHg)			0.69	0.00*
Diastolic Blood Pressure (mmHg)	0.67	0.00*		
Pulse rate (BPM)	0.03	0.87	0.39	0.64
Waist circumference (m)	−0.18	0.36	−0.23	0.23
Hip Circumference (m)	−.019	0.31	−0.19	0.32
Waist/hip ratio	−0.05	0.79	−0.16	0.41
Fat (kg)	−0.03	0.88	−0.09	0.65
Lean tissue (kg)	0.00	0.99	0.11	0.58
Lean Tissue and Bone Mineral Content (kg)	−.018	0.92	0.09	0.62
Total Mass (kg)	0.56	0.77	0.04	0.85
Fat (%)	0.06	0.78	−0.11	0.57
Lean (%)	0.06	0.74	0.13	0.51
Insulin Sensitivity (X10^−4^ min^−1^/μU/ml)	0.30	0.12	0.26	0.18
Acute Insulin Response (μU/ml X 10 min)	−0.03	0.99	0.08	0.67
Disposition Index (X10^−4^ min^−1^)	−0.33	0.09	−0.17	0.39
Fasting Glucose (mg/dl)	0.12	0.53	0.06	0.77
CRP (ng/ml)	−0.12	0.56	−0.02	0.32
Adiponectin (μg/ml)	0.44	0.02	0.41	.020*

**Table 3:** Multiple linear regression analysis for SBP

	Model 1	Model 2	Model 3	Model 4	Model 5	Model 6
Adiponectin (μg/ml)	0.439±0.061 0.022	0.472±0.060 0.013	0.462±0.063 0.021	0.488± 0.065 0.019	0.350±0.075 0.127	0.308± 0.77 0.189
BMI (kg/m2)		0.273±0.331 0.134				
Waist Circumference (cm)			−0.043±0.188 0.817			
Fasting Glucose (mg/dl)				−0.118±0.168 0.553		
Insulin Sensitivity (X10^−4^ min^−1^/μU/ml)					0.278±1.285 0.227	
Disposition Index (X10^−4^ min^−1^)						−0.236±−.003 0.378

The regression coefficient (β±SE) and P value are indicated.

**Table 4:** Multiple linear regression analysis for DPB

	Model 1	Model 2	Model 3	Model 4	Model 5	Model 6
Adiponectin (μg/ml)	0.428±0.047 0.026	0.441±0.048 0.025	0.408±0.05 0.045	0.401±0.052 0.06	0.348±0.061 0.159	0.324±0.064 0.021
BMI (kg.m2)		0.110±0.265 0.558				
Waist Circumference (m)			−0.130±0.149 0.505			
Fasting Glucose (mg/dl)				0.033±0.135 0.868		
Insulin Sensitivity (X10^−4^ min^−1^/μU/ml)					0.107±1.061 0.662	
Disposition Index (X10^−4^ min^−1^)						0.324±0.64 0.207

The regression coefficient (β±SE) and P value are indicated.

## Data Availability

The data for this study is available upon written request. For data requests please contact Dr. Louise Kelly, lakelly@callutheran.edu

## What is known about this subject

The prevalence of hypertension in children is threefold higher in obese than non-obese children. Furthermore, the cytokine adiponectin has been associated with the regulation of blood pressure (BP) in adults and adolescents. Adiponectin has been shown to be associated with Type 2 diabetes and the metabolic syndrome.

## What this study adds:

Adiponectin was positively and significantly correlated to blood pressure in obese Latino adolescent youth. To our knowledge, we are the first group to investigate the relationship between adiponectin and blood pressure in obese Latino adolescent boys with a family history of type 2 diabetes.

## Abbreviations

BP: Blood Pressure
SBP: Systolic Blood Pressure
DBP: Diastolic Blood pressure
